# IgG4‐related retroperitoneal fibrosis induced by nivolumab and ipilimumab in a patient with non‐small cell lung cancer: A case report

**DOI:** 10.1111/1759-7714.15155

**Published:** 2023-12-14

**Authors:** Masashi Nishimura, Yoshifumi Kimizuka, Takunori Ogawa, Motohiro Tsuchiya, Yoshiki Kato, Akira Matsukida, Shunya Igarashi, Koki Ito, Yusuke Serizawa, Tomomi Tanigaki, Yuji Fujikura, Yuka Katsurada, Sho Ogata, Akihiko Kawana

**Affiliations:** ^1^ Division of Infectious Diseases and Respiratory Medicine, Department of Internal Medicine National Defense Medical College Saitama Japan; ^2^ Department of Pathology and Laboratory Medicine National Defense Medical College Saitama Japan

**Keywords:** IgG4‐related disease, immune‐related adverse event, ipilimumab, nivolumab, retroperitoneal fibrosis

## Abstract

IgG4‐related diseases are adverse events that occur after receiving treatment with immune checkpoint inhibitors (ICI). This study reports the first case of IgG4‐related retroperitoneal fibrosis after the administration of chemotherapy with nivolumab and ipilimumab (NI therapy). An 80‐year‐old man developed lower abdominal pain eight months after NI therapy was initiated. Although the primary lesion maintained its reduced size on computed tomography, there was an increase in the soft tissue shadows intensity around the abdominal aorta, bladder, and seminal vesicles, suggesting retroperitoneal fibrosis. Blood tests showed elevated IgG4 levels. Computed tomography‐guided biopsy of the retroperitoneum showed B cell‐dominant lymphocyte infiltration consistent with IgG4‐related retroperitoneal fibrosis and characteristic CD8‐positive lymphocyte infiltration, suggestive of the involvement of cytotoxic T cells. Based on the clinical, imaging, and pathological findings, the patient was diagnosed with IgG4‐related retroperitoneal fibrosis due to ICI. Immunotherapy discontinuation alone did not result in improvement; therefore, steroid therapy was initiated. In clinical practice, IgG4‐related retroperitoneal fibrosis can occur as an immune‐related adverse event when administering anti‐PD‐1 and anti‐CTLA‐4 antibodies for cancer immunotherapy. Early steroid therapy could be effective in controlling this immune‐related adverse event.

## Graphical abstract

This case report describes a patient with IgG4‐related retroperitoneal fibrosis as an immune‐related adverse event when administering anti‐PD‐1 and anti‐CTLA‐4 antibodies for cancer immunotherapy. Immunotherapy discontinuation alone did not result in improvement. Systemic steroid therapy was initiated, and imaging showed reduction in size of the retroperitoneal fibrosis.

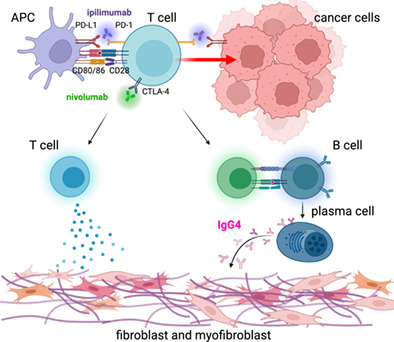

## INTRODUCTION

IgG4‐related adverse events, such as retroperitoneal fibrosis, occur after the patients receive treatment with immune checkpoint inhibitors (ICI).^1^, ^2^ Retroperitoneal fibrosis is etiologically classified as idiopathic or secondary. Previous studies reported secondary retroperitoneal fibrosis due to the administration of the PD‐1 antibody, nivolumab monotherapy, in ICI‐related cases; however, to our knowledge, no pathological evidence exists for IgG4‐related disease, such as retroperitoneal fibrosis as an irAE.^3^ Therefore, we report the first case of IgG4‐related retroperitoneal fibrosis induced by NI, supported by imaging and pathological findings.

## CASE REPORT

**FIGURE 1:**
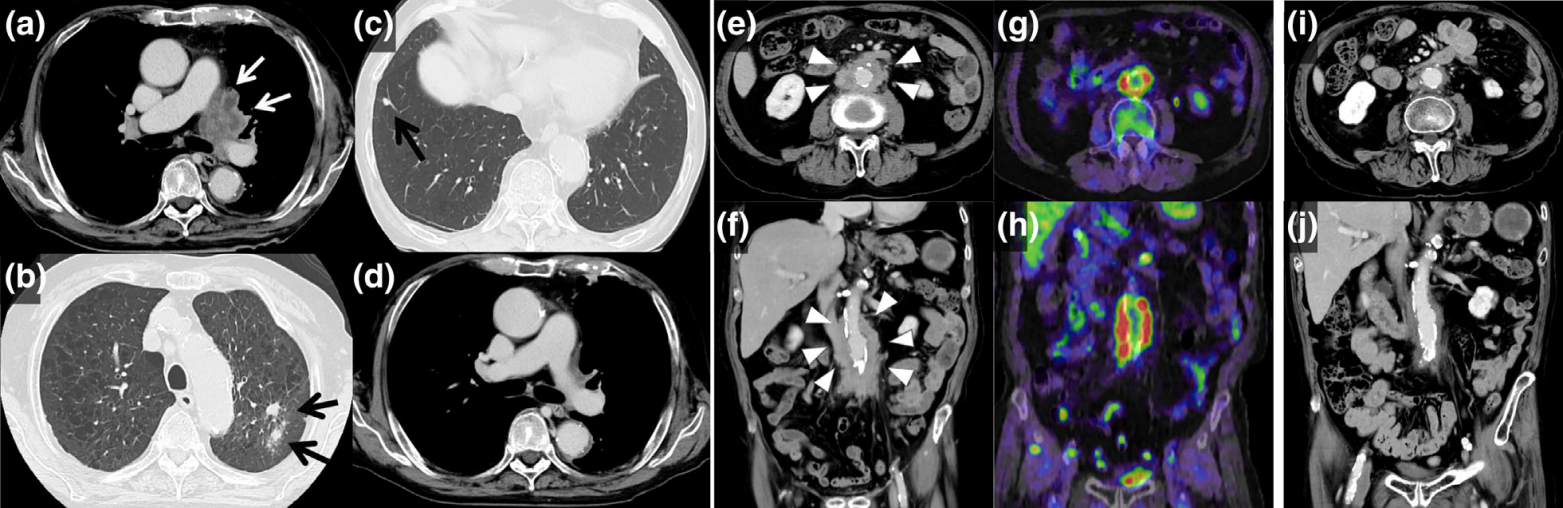
Imaging studies of the chest with the relevant timeline. (a–c) Chest contrast‐enhanced computed tomography (CT) scan revealed a tumor lesion that was fused between the left hilum and mediastinum (white arrow). The tumor enveloped the left pulmonary artery, and nodules were detected in both lung fields (black arrow). (d) A chest contrast‐enhanced CT scan taken eight months after the initiation of treatment showed a reduction in the tumor size around the left pulmonary artery. (e: cross‐section, f: coronal‐section) A pelvic contrast‐enhanced CT scan taken at the same time as that of (d) revealed soft tissue shadows around the abdominal aorta that had increased in size (arrowheads). Soft tissue shadows around the bladder and seminal vesicles had also increased in size. (g: cross‐section, h: coronal‐section) A positron‐emission tomography (PET‐CT) scan taken at the same time as that of (d) showed accumulation around the abdominal aorta and bladder. (i: cross‐section, j: coronal‐section) One month after the initiation of steroid treatment, a CT scan showed a reduction in the soft tissue shadow around the abdominal aorta.

An 80‐year‐old male developed hoarseness in October 2021 and visited the Otolaryngology Department on November 15, 2021. Chest computed tomography (CT) revealed a mass in the left upper lobe, multiple nodules, mediastinal lymph node enlargement, and pleural thickening (Figure 1a–c). He was diagnosed with lung adenocarcinoma based on lung tumor biopsy following thoracoscopic surgery. Molecular biology testing did not detect driver gene mutation/translocation and the PD‐L1 tumor proportion score (TPS) was <1%. The tumor stage was cT4N3M1a (stage IV A). Immunotherapy with nivolumab (360 mg/bodyweight) and ipilimumab (1 mg/kg) was initiated on February 1, 2022 for advanced cell lung cancer with PD‐L1 level <1%. Eight months later, the primary lesion size had reduced in size (Figure 1d).

**FIGURE 2:**
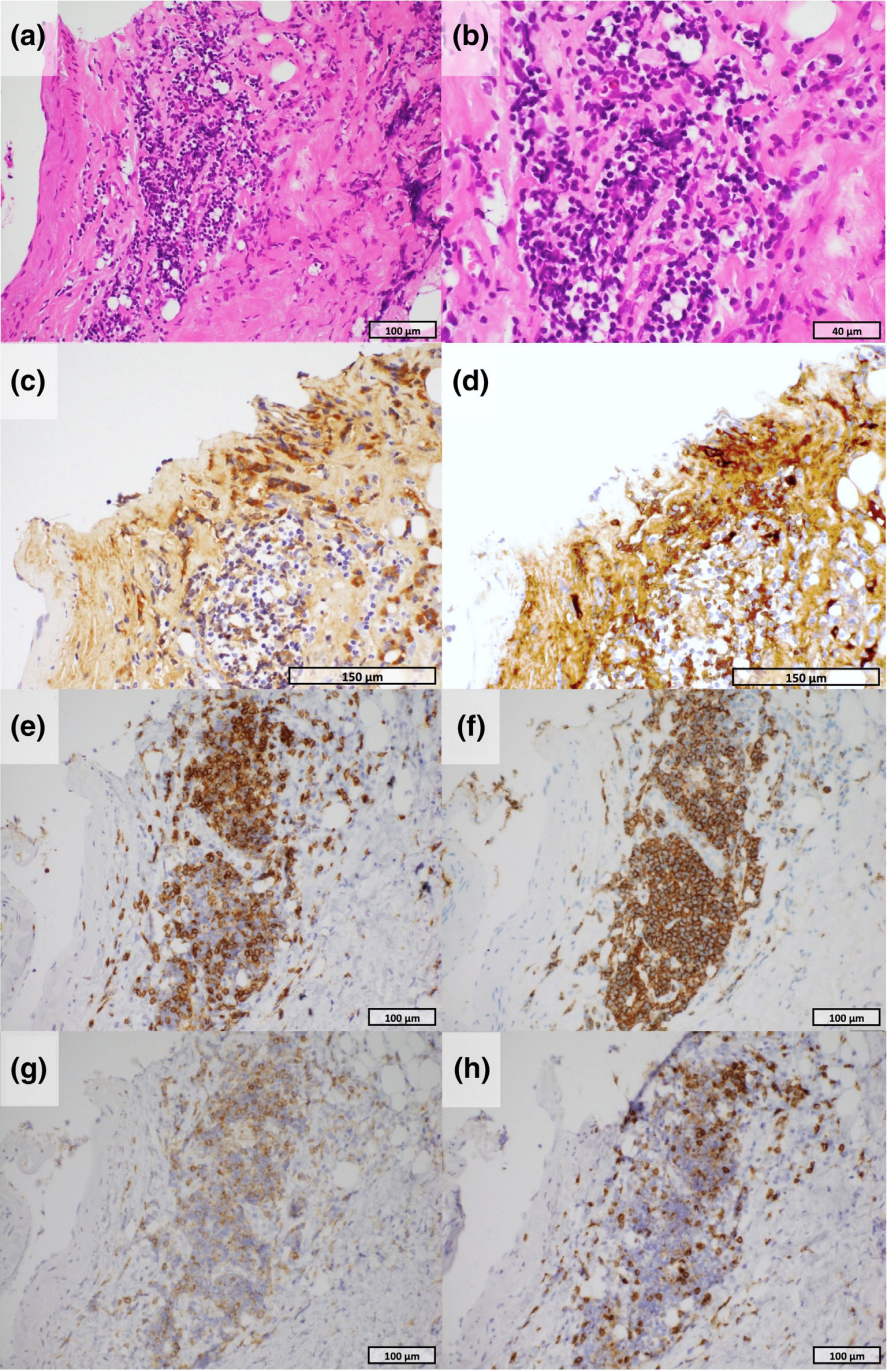
Pathological findings of retroperitoneal tissue (a, b: hematoxylin and eosin staining). Microscopic examination revealed lymphocytic infiltration with layered fibrosis (a: 40×, b: 400×) (c: immunohistochemistry [IHC] for IgG). IHC staining demonstrated the presence of IgG‐positive plasma cells (×100) (d: IHC for IgG4). IHC staining revealed the presence of numerous IgG4‐positive plasma cells. The number of IgG4‐positive cells corresponded to approximately 70% of the total number of IgG‐positive cells in the same area (100×) (e: IHC for CD3, f: IHC for CD20). Immunohistochemistry demonstrated that lymphocytes were positive for both CD3 (e: ×100) and CD20 (f: ×100), suggesting an association with both T and B cell lymphocytes (g: IHC for CD4, h: IHC for CD8). T cell lymphocytes infiltrating the lesion were positive for both CD4 and CD8 with a predominance of CD8.

The patient subsequently developed lower abdominal pain in late October 2022. The CT scan revealed an increased change in the soft tissue shadow around the abdominal aorta, bladder, and seminal vesicles, suggesting retroperitoneal fibrosis (Figure 1e, f). Positron emission tomography (PET‐CT) revealed fluorodeoxyglucose (FDG) accumulation in the same area (Figure 1g,h). Blood tests revealed a decrease in the CEA levels (CEA, 173.5 to 3.5 ng/mL), but an increase in the IgG4 levels (IgG4, 132 to 250 mg/dL; normal range, 80–140 mg/dL). Antinuclear antibodies were positive at a low titer of 1/40, whereas ACE and antineutrophil cytoplasmic antibodies were negative (Table 1). CT‐guided biopsy of the thickened retroperitoneum performed on December 6 showed infiltration of lymphocytes and morphologically‐abnormal cells, but storiform fibrosis and obliterative phlebitis were unremarkable. Immunohistochemical analysis revealed approximately 145 IgG4‐positive cells per high‐power field, accounting for approximately 71% of the IgG‐positive cells at the same site. The infiltrating lymphocytes were mainly CD20‐ and CD79a‐positive B cells, and both CD4‐ and CD8‐positive T cells were observed, with CD8 being predominantly present, but no malignancy was observed (Figure 2). Accordingly, the patient was diagnosed with IgG4‐related retroperitoneal fibrosis. The patient was observed after discontinuing immunotherapy. One month later, the serum IgG4 level remained high (IgG4, 252 mg/dL), and enlargement of the soft tissue shadow was observed on CT scan; therefore, systemic steroid therapy was initiated (prednisolone, 50 mg/day, 1 mg/kg/day). One month later, imaging showed a reduction in size of the adipose tissue around the abdominal aorta and bladder (Figure 1i,j). Thereafter, the systemic steroid dose was gradually reduced and no worsening of the malignant tumor or retroperitoneal fibrosis was observed.

**TABLE 1:** Results of blood tests before treatment administration.

Hematology		Biochemistry			
White blood cells	6800/uL	T‐Bil	0.36 mg/dL	CRP	1.5 mg/dL
Neutrophil	46.9%	AST	15 IU/L	IgG	1849 mg/dL
Lymphocyte	35.6%	ALT	11 IU/L	IgA	1113 mg/dL
Basophil	0.4%	LDH	167 IU/L	IgM	39 mg/dL
Eosinophil	10.6%	TP	7.6 g/dL	IgG4	250 mg/dL
Monocyte	6.5%	Alb	3.2 g/dL	ACE	15.8 IU/L
Red blood cells	405×10^4^/mL	BUN	16 mg/dL	PR3‐ANCA	<1.0 IU/mL
Hemoglobin	10.9 g/dL	Cr	0.97 mg/dL	MPO‐ANCA	<1.0 IU/mL
Hematocrit	33.7%	Na	137 mEq/L	ANA	40 × (Speckled)
Platelets	40×10^4^/mL	K	4.1 mEq/L	CH50	63.9 IU/mL
		Cl	104 mEq/L	C3	42 mg/dL
				C4	40 mg/dL

Abbreviations: ACE, angiotensin‐converting enzyme; ANA, antinuclear antibody; PR‐3/MPO‐ANCA, proteinase‐3/ myeloperoxidase antineutrophil cytoplasmic antibody.

## DISCUSSION

The pathological findings of idiopathic retroperitoneal fibrosis are typically observed as hardened tissue mixed with mononuclear cells, with an increase in fibroblasts and collagen fibers accompanying a vigorous chronic inflammation with numerous lymphocytes, plasma cells, and macrophages.^4^ Typically, CD20‐positive B cells outnumber CD4‐positive T cells.^4^ In this report, immunohistochemically, scattered clusters of CD20‐ and CD79a‐positive cells were observed, and B cells were predominantly recognized, which is consistent with the known pathological findings of IgG4‐related retroperitoneal fibrosis. However, a characteristic finding of IgG4‐related retroperitoneal fibrosis was the infiltration of CD8‐positive T lymphocytes into the tissue, which was more prominent than that of CD4‐positive cells. The mechanism of irAE development comprises the involvement of activated B cells in the production of autoantibodies due to T cells activation and the increase in inflammatory cytokines.^5^ Moreover, it is assumed that PD‐1/PD‐L1 pathway inhibition, by promoting the growth and differentiation of B cells, and class switching to IgG4, by promoting T follicular helper cells, induces the disease. However, the pathological tissue implies the involvement of a significant number of cytotoxic T cells in the pathogenesis, which is atypical for IgG4‐related diseases and might indicate the specific pathology of ICI‐induced IgG4‐related retroperitoneal fibrosis.

Treatment for irAEs typically involves discontinuing ICIs for patients with grade 2 followed by steroid treatment if no improvement is observed. For patients with grade 3 or higher, ICI discontinuation followed by steroid treatment is pursued.^6^ In our case, observation was initially attempted through ICI discontinuation, but due to the worsening of retroperitoneal fibrosis, steroid treatment was initiated. It is hypothesized that steroid therapy not only suppressed the function and activity of T cells, which is central to the immunological mechanism of irAE, but also reduced the levels of B cells and immunoglobulins, which could have had an effect on controlling the ICI‐induced IgG4‐related retroperitoneal fibrosis.

In the clinical setting, retroperitoneal fibrosis due to IgG4‐related diseases can occur when administering anti‐PD‐1 and anti‐CTLA‐4 antibodies for cancer immunotherapy. Early steroid treatment is important for controlling irAEs and IgG4‐related retroperitoneal fibrosis by ICIs.

## AUTHOR CONTRIBUTIONS

Masashi Nishimura: Resources, conceptualization, writing – original draft, visualization. Yoshifumi Kimizuka: Conceptualization, writing – original draft, visualization, project administration. Takunori Ogawa: Conceptualization, visualization, data curation. Motohiro Tsuchiya: Visualization, formal analysis, data curation. Yoshiki Kato: Resources. Akira Matsukida: Resources. Shunya Igarashi: Resources. Koki Ito: Resources. Yusuke Serizawa: Resources. Tomomi Tanigaki: Resources. Yuji Fujikura: Resources. Yuka Katsurada: Supervision. Sho Ogata: Supervision. Akihiko Kawana: Supervison.

## CONFLICT OF INTEREST STATEMENT

None of the authors have any conflicts of interest to disclose.

## INFORMED CONSENT STATEMENT

All authors confirm that they have obtained informed consent to publish information and images in this case report.

## References

[1] Terashima T , Iwami E , Shimada T , Kuroda A , Matsuzaki T , Nakajima T , et al. IgG4‐related pleural disease in a patient with pulmonary adenocarcinoma under durvalumab treatment: a case report. BMC Pulm Med. 2020;5:248–253. 10.1186/s12890-020-1150-x PMC7183131 32334571

[2] Sánchez‐Oro R , Alonso‐Mun õz EMLMR . Review of IgG4‐related disease. Gastroenterol Hepatol (N Y). 2019;42:638–647. 10.1016/j.gastrohep.2019.08.009 31722794

[3] Moreau A , Giraudet AL , Mognetti T , Kryza D . Retroperitoneal fibrosis in on‐going anti‐PD‐1 immunotherapy detected with [18F]‐FDG PET/CT. Eur J Nucl Med Mol Imaging. 2019;46:1758–1759. 31093709 10.1007/s00259-019-04352-1

[4] Vaglio A , Salvarani C , Buzio C . Retroperitoneal fibrosis. Lancet. 2006;35:241–251. 10.1016/S0140-6736(06)68035-5 16427494

[5] Mahajan VS , Mattoo H , Deshpande V , Pillai SS , Stone JH . IgG4‐related disease. Annu Rev Pathol. 2014;9:315–347. 24111912 10.1146/annurev-pathol-012513-104708

[6] Schneider BJ , Naidoo J , Santomasso BD , Lacchetti C , Adkins S , Anadkat M , et al. Management of immune‐related adverse events in patients treated with immune checkpoint inhibitor therapy: ASCO guideline update. J Clin Oncol. 2021;39:4073–4126. 34724392 10.1200/JCO.21.01440

